# Synthetic Cathinones Induce Cell Death in Dopaminergic SH-SY5Y Cells via Stimulating Mitochondrial Dysfunction

**DOI:** 10.3390/ijms21041370

**Published:** 2020-02-18

**Authors:** Huey Sze Leong, Morgan Philp, Martin Simone, Paul Kenneth Witting, Shanlin Fu

**Affiliations:** 1 Centre for Forensic Science, School of Mathematical and Physical Sciences, University of Technology Sydney, Ultimo, NSW 2007, Australia; huey.s.leong@student.uts.edu.au (H.S.L.); morgan.philp@uts.edu.au (M.P.); 2 Discipline of Pathology, Charles Perkins Centre, School of Medical Sciences, Faculty of Medicine and Health, The University of Sydney, Camperdown, NSW 2006, Australia; msim6013@uni.sydney.edu.au

**Keywords:** mitochondrial dysfunction, calcium dysregulation, apoptosis, synthetic cathinones, SH-SY5Y

## Abstract

Increasing reports of neurological and psychiatric complications due to psychostimulant synthetic cathinones (SCs) have recently raised public concern. However, the precise mechanism of SC toxicity is unclear. This paucity of understanding highlights the need to investigate the in-vitro toxicity and mechanistic pathways of three SCs: butylone, pentylone, and 3,4-Methylenedioxypyrovalerone (MDPV). Human neuronal cells of SH-SY5Y were cultured in supplemented DMEM/F12 media and differentiated to a neuronal phenotype using retinoic acid (10 μM) and 12-*O*-tetradecanoylphorbol-13-acetate (81 nM). Trypan blue and lactate dehydrogenase assays were utilized to assess the neurotoxicity potential and potency of these three SCs. To investigate the underlying neurotoxicity mechanisms, measurements included markers of oxidative stress, mitochondrial bioenergetics, and intracellular calcium (Ca^2+^), and cell death pathways were evaluated at two doses (EC_15_ and EC_40_), for each drug tested. Following 24 h of treatment, all three SCs exhibited a dose-dependent neurotoxicity, characterized by a significant (*p* < 0.0001 vs. control) production of reactive oxygen species, decreased mitochondrial bioenergetics, and increased intracellular Ca^2+^ concentrations. The activation of caspases 3 and 7 implicated the orchestration of mitochondrial-mediated neurotoxicity mechanisms for these SCs. Identifying novel therapeutic agents to enhance an altered mitochondrial function may help in the treatment of acute-neurological complications arising from the illicit use of these SCs.

## 1. Introduction

Synthetic cathinones (SCs) have emerged as popular supplements to “traditional” drugs of abuse, such as cocaine, 3,4-methylenedioxymethamphetamine (MDMA, Ecstasy or “Molly”), and methamphetamine (meth), due to their psychostimulant and entactogenic effects [1]. These illicit psychostimulants, often labeled as “not for human consumption”, are also marketed as “legal highs”, “bath salts”, “plant food”, and “research chemicals” to evade scrutiny and detection by law enforcement agencies. Their relatively low cost, ease of internet purchases, and the lack of control over clandestine drug manufacturers have greatly contributed to the explosion of these drugs in the recreational drug scene [2].

**Figure 1:**
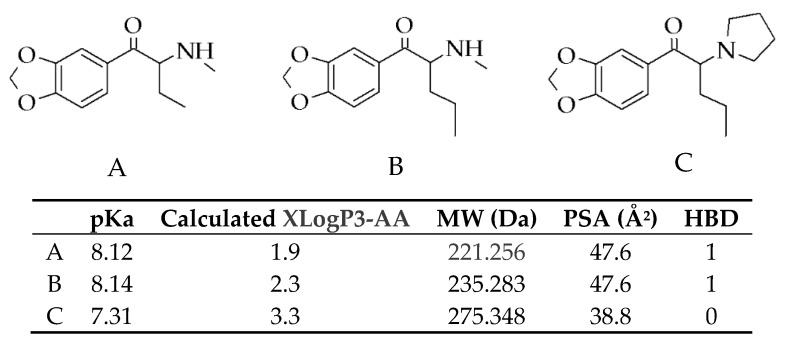
Chemical structures of three synthetic cathinones (SCs): (**A**) butylone, (**B**) pentylone, and (**C**) 3,4-Methylenedioxypyrovalerone (MDPV). Table: Estimated pKa values (measure of ionization) calculated using MarvinSketch software (https://chemicalize.com/), XLogP3-AA (measure of lipophilicity), molecular weight (MW), polar surface area (PSA), and hydrogen bond donors (HBD) of (**A**) butylone, (**B**) pentylone, and (**C**) MDPV. Data were obtained from the PubChem Compound Database (https://www.ncbi.nlm.nih.gov/pccompound/56843046; 60208608; 20111961). Optimal penetration of BBB: XLogP3-AA ranged from 1 to 4, MW < 450 Da, PSA < 90 Å^2^, and HBD < 3.

3,4-Methylenedioxypyrovalerone (MDPV) (chemical structure shown in Figure 1) is one of the most prevalent SCs found on the illegal drug market [3], with butylone and pentylone (Figure 1) being identified as substitute drugs among the “legal highs” [4,5,6,7,8,9]. Powders, pills, and tablets sold as “Molly” to electronic dance music attendees have also been found to be adulterated with butylone and pentylone [10,11,12].

SCs act as central nervous stimulants via escalating neurotransmitters’ concentration. Butylone and pentylone display hybrid transporter activity, characterized by inhibition effects at the dopamine transporter, DAT (more potent for pentylone), but substrate effects at the serotonin transporter, SERT (more potent for butylone) [13]. MDPV, on the other hand, is known for its potent inhibition effects at DAT and NET, but not as a substrate releaser [14]. SCs are readily self-administered, with pentylone and MDPV exhibiting a higher reinforcing efficacy, suggesting considerable potential for compulsive use [15,16].

The abuse of SCs poses serious public health concerns due to their high addiction potential and increasing number of fatalities [17,18]. Severe intoxications and death cases have been linked to the usage of butylone, pentylone, and MDPV [19,20,21,22,23,24,25], and the majority of cases have been connected to neuropsychiatric and neurological complications associated with SC toxicity [26,27]. In addition to the direct SC neurotoxicity effects, potential interactions, such as hyperthermia, toxic metabolites, excitoxicity, and neuroinflammation, may also contribute to SC-induced neurotoxicity [28]. In this study, the primary focus will be on the direct neurotoxicity effects that are potentiated by dopaminergic neuron signaling. Preclinical studies have suggested the potential neuronal damage induced by SCs [29]. Studies have also documented neurotoxic effects of synthetic cathinones in utero and early life exposure [30,31]. More recently, the repeated binge-like administration of MDPV in rats showed evidence of neurodegeneration [32]. Previous in-vitro studies have revealed the neurotoxic potential of several SCs [33,34,35,36,37,38], but the neurotoxicity potential of butylone and pentylone has remained unexplored. In recent years, oxidative stress and mitochondrial dysfunction mechanisms have been identified as significant contributors to SC-induced neurotoxicity [36,37,39,40]. However, the role of neuronal calcium signaling in mitochondrial dysfunction and the induction of neurotoxic cascades for SCs is scarce. The paucity of scientific research and understanding of neurotoxic mechanisms determined the aims of the study.

The present study aimed to evaluate the neurotoxicity potential and potency of butylone and pentylone in relation to MDPV using the well-established SH-SY5Y cell line that can be differentiated into a dopaminergic neuronal phenotype [41]. It also aimed to define the role of these SCs in promoting oxidative stress and mitochondrial dysfunction and altering calcium (Ca^2+^) levels as a plausible mechanistic pathway of neurotoxicity in cultured neurons.

## 2. Results

### 2.1. Butylone, Pentylone, and MDPV Elicited Neurotoxicity with Different Potencies

**Figure 2:**
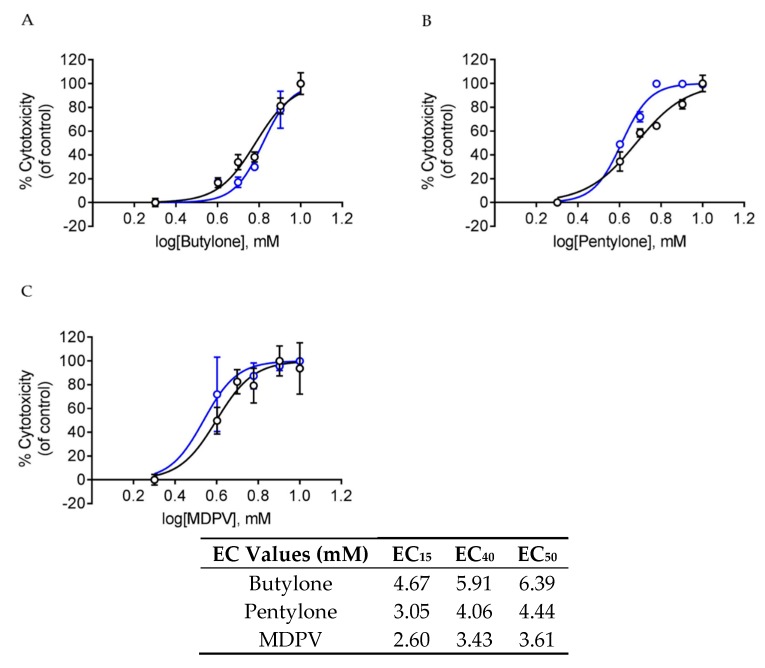
Dose-response curves after the treatment of (**A**) butylone, (**B**) pentylone, and (**C**) MDPV for 24 h; lactate dehydrogenase (LDH) (black) and trypan blue (TB) assay (blue). Data are Mean ± SD obtained from three independent experiments for the TB assay and four independent experiments for the LDH assay. Table: Mean EC_15_, EC_40_, and EC_50_ values after 24 h of treatment of butylone, pentylone, and MDPV, obtained from both TB and LDH assays.

After 24 h of drug treatment, all of the SCs displayed a dose-dependent toxicity, whereby increasing the drugs’ dosage increased the percentage of cell death of dopaminergic neuronal SH-SY5Y cells (Figure 2A–C). Both the trypan blue (TB) assay and lactate dehydrogenase (LDH) assay produced similar dose-response curves and EC_50_ values for all SCs. Butylone and pentylone had higher EC_50_ values when compared to MDPV. In order to elucidate the possible mechanisms underlying the SC-elicited neurotoxicity, EC_15_ and EC_40_ doses were chosen for subsequent studies.

### 2.2. Butylone, Pentylone, and MDPV Triggered Oxidative Stress

**Figure 3:**
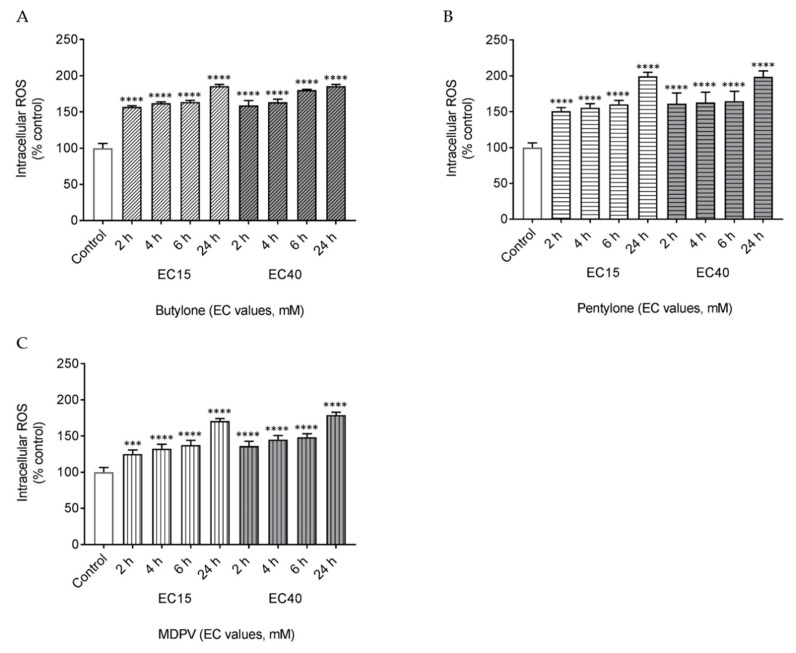
Intracellular levels of reactive oxygen species (ROS) after 2, 4, 6, and 24 h treatment of EC_15_ and EC_40_ for (**A**) butylone, (**B**) pentylone, and (**C**) MDPV. Data are Mean ± SD obtained from three independent experiments. Different to the control; **** *p* < 0.0001 and *** *p* < 0.001.

To determine the role of oxidative stress in SC-induced neurotoxicity, the effect of butylone, pentylone, and MDPV on the production of intracellular reactive oxygen species (ROS) was examined at different time points (2, 4, 6, and 24 h). All the SCs demonstrated a time-dependent increased ROS production starting as early as 2 h post drug treatment. At 24 h, both EC_15_ and EC_40_ doses of butylone, pentylone, and MDPV induced approximately a two-fold increase of ROS production (Figure 3A–C).

### 2.3. Butylone, Pentylone, and MDPV Compromised Mitochondrial Bioenergetics

**Figure 4:**
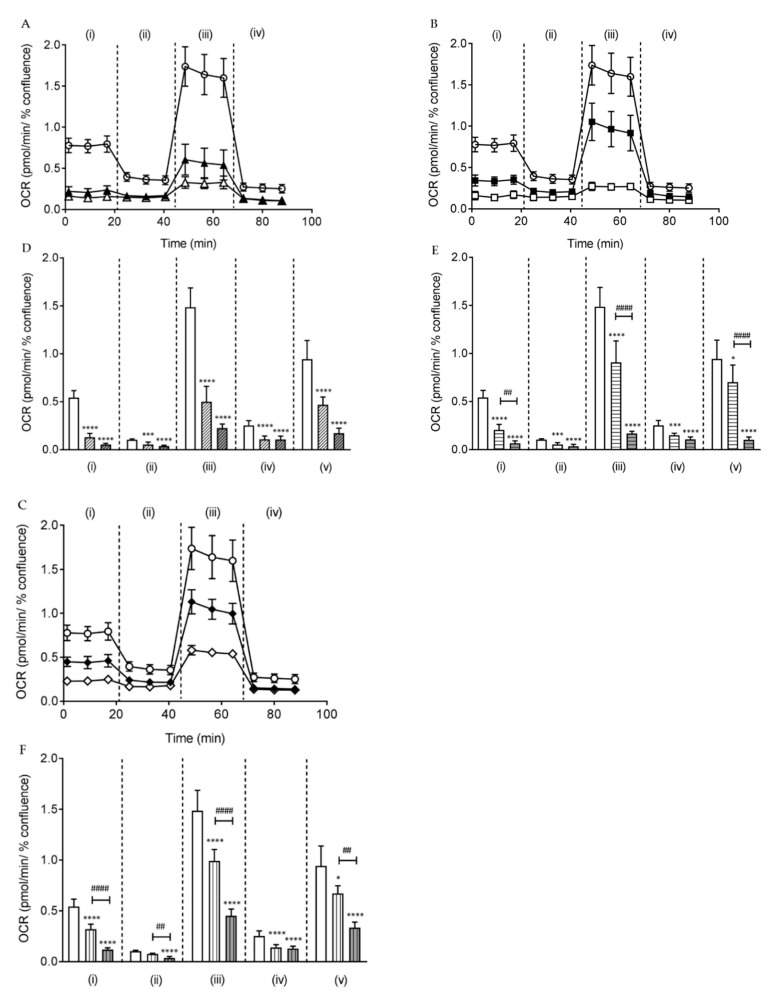
Oxygen consumption rate (OCR) measurement over time after 24 h of treatment of EC_15_ and EC_40_ for (**A**) butylone (filled and empty triangle, respectively), (**B**) pentylone (filled and empty square, respectively), and (**C**) MDPV (filled and empty diamond, respectively) with control (empty circle). Individual mitochondrial function parameters of EC_15_ and EC_40_ for (**D**) butylone (clear and grey diagonal bars, respectively), (**E**) pentylone (clear and grey horizontal bars, respectively), and (**F**) MDPV (clear and grey vertical bars, respectively): (i) basal respiration (^##^ *p* < 0.01 vs. EC_15_ pentylone, and ^####^ *p* < 0.0001 vs. EC_15_ MDPV), (ii) proton leak (^##^ *p* < 0.01 vs. EC_15_ MDPV), (iii) maximal respiration (^####^ *p* < 0.0001 vs. EC_15_ pentylone and EC_15_ MDPV), (iv) non-mitochondrial respiration, and (v) spare respiratory capacity (^####^ *p* < 0.0001 vs. EC_15_ pentylone, and ^##^ *p* < 0.01 vs. EC_15_ MDPV). The spare respiratory capacity was calculated from the difference between maximal and basal respiration. Data are Mean ± SD obtained from at least four independent experiments normalized to the % confluence of cells. Different to the control; * *p* < 0.1, *** *p* < 0.001 and **** *p* < 0.0001.

The production of ROS is often linked to mitochondrial respiratory chain dysfunction. The Optimized Seahorse Mito Stress assay was used to assess key parameters of mitochondrial bioenergetics by measuring the oxygen consumption rate (OCR) of cells. This was performed by sequential compound injections that targeted components of the electron transport chain (ETC) in the mitochondria. All of the SCs reduced mitochondrial respiration (expressed by OCR) and increased mitochondrial stress levels in cultured SH-SY5Y cells relative to the control at both doses EC_15_ and EC_40_ after 24 h of drug treatment (Figure 4A–F). The OCR for basal and maximal respiration decreased markedly (**** *p* < 0.0001 vs. control) and the decreased OCR was found to be dose-dependent for pentylone and MDPV. The OCR for proton leak and non-mitochondrial respiration was also found to decrease significantly when cultured SH-SY5Y cells were treated with the EC_40_ dose (**** *p* < 0.0001 vs. control). Furthermore, the OCR for the spare respiratory capacity reduced markedly at the EC_40_ dose (**** *p* < 0.0001 vs. control) and was dose-dependent for pentylone and MDPV. Collectively, these findings show that all three SCs at EC_40_ doses profoundly impaired the mitochondrial function by the disruption of respiration in dopaminergic neuronal SH-SY5Y cells.

**Figure 5:**
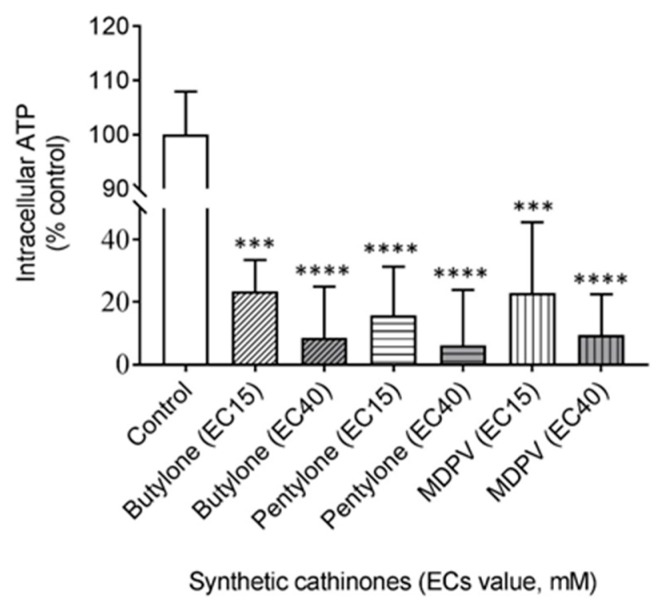
Intracellular levels of adenosine triphosphate (ATP) after 24 h of treatment of EC_15_ and EC_40_ for butylone, pentylone, and MDPV. Data are Mean ± SD obtained from three independent experiments normalized to total protein. Different to the control; *** *p* < 0.001 and **** *p* < 0.0001.

Impairment of the mitochondrial ETC function often leads to a subsequently compromised bioenergetics balance. To further confirm that butylone-, pentylone-, and MDPV-induced mitochondrial inhibition cause the impairment of cellular bioenergetics, a highly sensitive luminescence-based assay was employed to assess the intracellular adenosine triphosphate (ATP) levels in cells. After 24 h of drug treatment, all of the SCs stimulated significant intracellular ATP depletion when cells were exposed to SCs at both EC_15_ and EC_40_ doses (all values significantly decreased relative to the control; 100% value corresponding to 11.4 mM/mg protein, Figure 5). Therefore, the residual ATP levels in cultured SH-SY5Y cells determined for butylone, pentylone, and MDPV (administered at their corresponding EC_15_ doses) were 23.5 ± 10.0%, 15.8 ± 15.5%, and 23.0 ± 22.5%, respectively. ATP levels were further reduced to 8.5 ± 16.5%, 6.2 ± 17.8%, and 9.5 ± 12.9% at EC_40_ doses for butylone, pentylone, and MDPV, respectively.

### 2.4. Butylone, Pentylone, and MDPV Altered Neuronal Ca^2+^ Homeostasis

**Figure 6:**
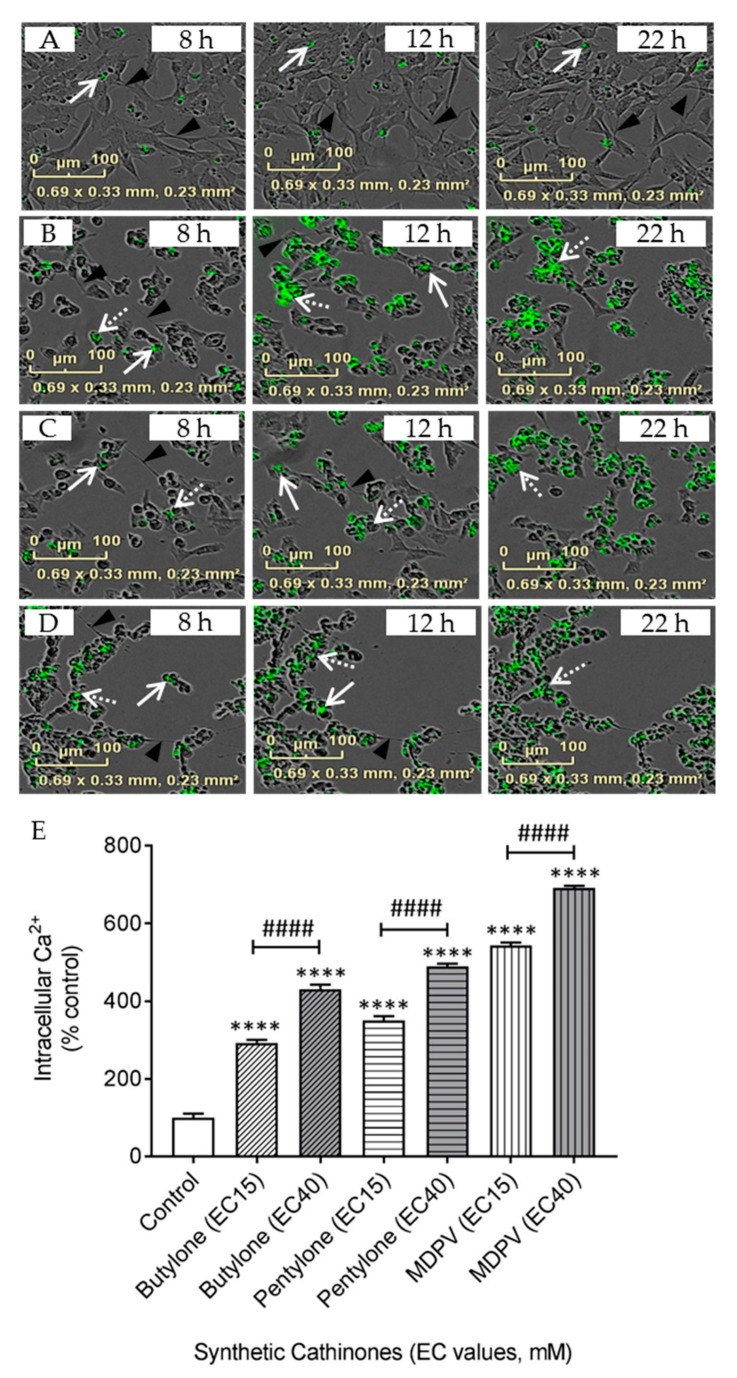
Representative merged images of Cal-520AM staining in (**A**) the control, (**B**) EC_15_ butylone, (**C**) EC_15_ pentylone, and (**D**) EC_15_ MDPV using the IncuCyte^®^ live-cell imaging system (scale bar: 100 μm) at different time points (8, 12, and 22 h) after drug treatments. Triangular arrowhead shows cell axons, the filled arrow shows the focal distribution, and the broken arrow shows the dispersed distribution of Cal-520 AM staining. (**E**) Quantification of Cal-520 AM fluorescence after 24 h of treatment of EC_15_ and EC_40_ for butylone, pentylone, and MDPV. Data shown in panel E are Mean ± SD obtained from four independent experiments. ^####^ *p* < 0.0001 vs. EC_15_ butylone, EC_15_ pentylone and EC_15_ MDPV. Different to the control; **** *p* < 0.0001.

Direct imaging and measurement of Ca^2+^ provides important insights into the regulation of intracellular Ca^2+^ in neurons. Treatment with drugs for 24 h significantly changed the neuronal phenotype of branched dendrites (triangular arrowhead, Figure 6A) to that of rounded shaped cells with neurite retraction (Figure 6B–D). In the controls (no drug treatment), a focal distribution of Ca^2+^ fluorescence was detected (filled arrow, Figure 6A), while a mixture of a focal and dispersed cytoplasmic Ca^2+^ distribution (broken arrow) was detected in cells treated with SCs at EC_15_ concentrations (Figure 6B–D). The dispersion of Ca^2+^ fluorescence was observed as early as 8 h after drug treatment, with an increasing Ca^2+^ fluorescence intensity occurring in a time-dependent manner and reaching a steady state between 12 and 22 h.

The quantification of intracellular Ca^2+^ showed that the intracellular Ca^2+^ levels increased significantly 24 h after drug treatment (Figure 6E). Intracellular Ca^2+^ levels in cells exposed to butylone, pentylone, and MDPV (administered at corresponding EC_15_ doses) were 292.3 ± 8.5%, 350.1 ± 11.0%, and 543.2 ± 7.6%, respectively. Intracellular Ca^2+^ was further increased to 430.7 ± 11.5%, 489.5 ± 6.8%, and 690.9 ± 5.9% at EC_40_ doses for butylone, pentylone, and MDPV, respectively. The increased intracellular Ca^2+^ concentrations were dose-dependent for all SCs (^####^ *p* < 0.0001 vs. EC_15_).

### 2.5. Butylone, Pentylone, and MDPV Induce an Apoptotic Cell Death Pathway

**Figure 7:**
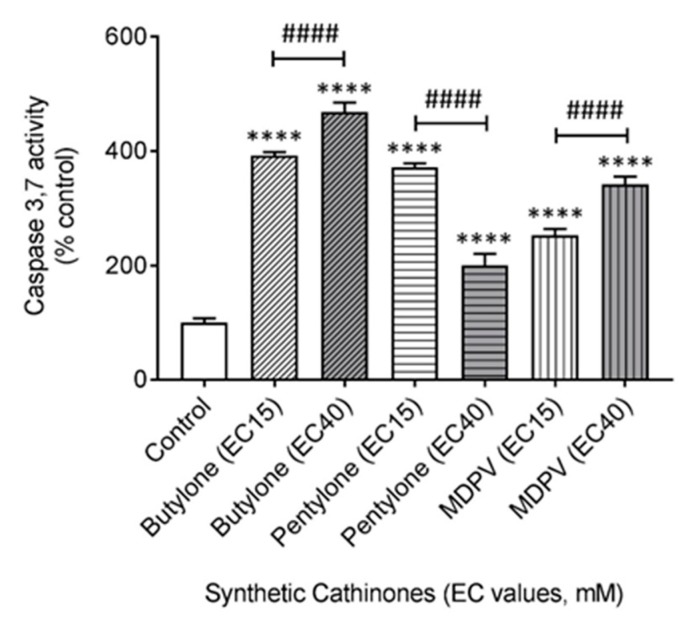
Effect elicited by EC_15_ and EC_40_ for butylone, pentylone, and MDPV in the activation of caspase 3 and 7 after 24 h of treatment. Data are Mean ± SD obtained from four independent experiments normalized to the % confluence of cells. ^####^ *p* < 0.0001 vs. EC_15_. Different to the control; **** *p* < 0.0001.

To investigate whether SC neurotoxicity leads to mitochondrial programmed cell death, measurement of the apoptosis executioner caspases 3 and 7 was performed. Caspase cleavage of the proluminescent substrate liberates free aminoluciferin that is consumed by luciferase to generate a glow-type luminescent signal. The signal produced is proportional to the caspase 3 and 7 activity. The administration of butylone, pentylone, and MDPV promoted caspase 3 and 7 activation in cultured SH-SY5Y cells 24 h after treatment (Figure 7). Caspase activation values in cells exposed to butylone, pentylone, and MDPV (administered at their corresponding EC_15_ doses) were 392.0 ± 6.2%, 372.0 ± 6.8%, and 253.1 ± 10.8% vs. the control, respectively. Caspase activation was further increased to 468.4 ± 16.7% and 342.0 ± 13.5% in cells treated with EC_40_ doses for butylone and MDPV, respectively, but decreased to 200.0 ± 20.5% at EC_40_ doses for pentylone. Both butylone and MDPV stimulated a dose-dependent increase in caspase activity (^####^ *p* < 0.0001 vs. EC_15_).

## 3. Discussion

The present findings highlight the potential neurotoxicity potency associated with the abuse of butylone, pentylone, and MDPV. The neurotoxicity of these SCs resulted in a raft of cellular and molecular level effects, including the accumulation of ROS production, mitochondrial dysfunction, and the alteration of Ca^2+^ homeostasis that finally led to apoptotic cell death. The increased incidence of neurological and psychiatric changes involving cognitive impairments and mental illnesses after SC use further demonstrated their neurotoxicity [42]. A growing number of studies have suggested that SCs induce neurocognitive dysfunction among acute and long-term users [32]. Currently, there is no antidote for acute intoxications of SCs, although behavioral-based and lifestyle interventions, together with seemingly promising pharmacotherapy drug treatment, are gaining more attention.

Our study demonstrates that SCs exert significant neurotoxic effects on cultured dopaminergic neuronal SH-SY5Y cells. The dose-dependent neurotoxicity pattern demonstrated showed the detrimental impact of these SCs upon exposure when administered. Overall, butylone appeared to be less potent when compared to pentylone and MDPV (EC_50_: butylone 6.39, pentylone 4.44, and MDPV 3.61 mM). The orders of drug potency determined by both TB and LDH assays were similar (MDPV ≈ pentylone > butylone). These results were consistent with previous studies conducted, where an increasing cytotoxicity was documented for drugs with longer alkyl side-chains. Such elongation is thought to increase the lipophilicity (XLogP3-AA values, Figure 1: butylone 1.9, pentylone 2.3, and MDPV 3.3) and hence its ability to penetrate the cell membrane, in order to facilitate cell death [43,44]. Drug potency is also influenced by drug permeability of the blood–brain barrier (BBB). All the SCs’ physicochemical properties were within the optimal ranges (Figure 1), suggesting the ease with which these SCs can cross the BBB [45,46] and exert their pharmacological effects on the monoamine transporters. However, the relative neurotoxic potencies (demonstrated by the difference in EC_50_ values) shown did not correlate with the values of previously reported DAT inhibition potencies [47,48], indicating that the neurotoxicity of butylone, pentylone, and MDPV seemed to be independent of DAT-mediated uptake. These findings are in agreement with an independent study conducted by Valente et al., whereby no protective effect of the GBR 12909 DAT inhibitor was observed for MDPV-induced neurotoxicity [36]. It is not known whether these SCs gain entry into cells via other carrier-mediated uptake or by diffusion, demonstrating that the precise underpinnings of cellular uptake are not fully understood and require future investigation.

The formation of toxic reactive oxygen and nitrogen species has been implicated in the promotion of neuronal cell death. Recently, there has been increasing evidence that ROS are responsible for SC-related neuronal damage. In this study, the rapid and time-dependent production of intracellular ROS (Figure 3) was observed, indicative of a central role for oxidative stress involvement in the neurotoxicity mediated by butylone, pentylone, and MDPV. ROS production following this SC exposure might be an early biochemical indicator that precedes neuronal cell death. It is unclear why ROS production under these experimental conditions showed a time-dependent, but not dose-dependent, increment for these SCs. However, the findings from this study are in agreement with recent in-vitro studies whereby oxidative stress was found to be involved in the mechanism of action of the SCs 4-Methylmethcathinone (4-MMC), 3,4-Methylenedioxymethcathinone (MDMC), α-Pyrrolidinooctanophenone (α-POP), α-Pyrrolidinononanophenone (α-PNP), methylone, 3-Fluoromethcathinone (3-FMC), and MDPV [36,37,39,40,43,49,50]. Although the mechanism underlying ROS production remains to be elucidated, it is clear that intracellular ROS promote oxidative stress and potentiate mitochondrial dysfunction in neuronal cells.

Mitochondria, the major regulators of cellular energy metabolism, have been recognized as the primary source of intracellular ROS and the key player in amphetamine-induced stress [51]. They act as energy generators in cells via oxidative phosphorylation and ATP production. Mitochondrial respiration impairment has been implicated in neuronal death and several neurodegenerative diseases. In the basal state, mitochondrial oxygen consumption is mainly driven by ATP synthase. Drug treatment at an EC_15_ dosage of butylone, pentylone, and MDPV led to a 41–76% inhibition of basal OCR in comparison to the control, suggesting significantly altered ATP consumption, even at low doses of SCs. Following the addition of oligomycin when evaluating the mitochondrial coupling, the OCR decreased and the remaining mitochondrial oxygen consumption was predominantly driven by the proton leak across the inner membrane. A substantial reduction of proton leak at EC_40_ doses (63–67% from the control), indicated that mitochondria were severely damaged and uncoupled. The addition of carbonyl cyanide 4-(trifluoromethoxy)phenylhydrazone, FCCP dissipated the proton gradient across the mitochondrial inner membrane, uncoupling electron transport from oxidative phosphorylation and hence facilitating measurement of the maximal OCR. A significant reduction of the maximal respiration capacity (33–66% from the control) signified dysfunction in the respiratory complexes and depletion of the mitochondrial membrane potential, which is crucial for the functional integrity of mitochondrial ETC. The final addition of rotenone and antimycin A shut down the mitochondrial ETC function, enabling the measurement of non-mitochondrial oxygen consumption. A significant reduction of non-mitochondrial oxygen consumption at both doses (41–58% from the control) implied an overall slowing down of cell metabolism due to energy stress. The spare respiratory capacity, the major determinant of the cells’ survival under the maximal physiological or pathophysiological condition, is a critical factor in maintaining the ATP-generating capacity reserve. At the EC_40_ dose, the level of OCR, a surrogate for the spare respiratory capacity, was reduced significantly (64–89% from the control), further confirming the inhibition of ETC by these SCs. Generally, the reduction of mitochondrial respiration demonstrated by the significantly decreased OCR of the individual parameters at EC_40_ doses (Figure 4A–F) compared to controls, indicated that butylone, pentylone, and MDPV may inhibit multiple components of the ETC, resulting in the impairment of mitochondria. Mitochondrial impairment upon MDPV exposure has been reported in SH-SY5Y cells using end-point measurement, such as mitochondrial membrane depolarization [36]. Up to now, there is only one other respiration kinetic study that has described the involvement of the mitochondrial inhibition of complexes within the ETC for SC-induced neurotoxicity [39]. In this study, the effects of butylone, pentylone, and MDPV were identified and quantified for impairment of the mitochondrial ETC function, leading to the mitochondrial dysfunction of dopaminergic neuronal SH-SY5Y cells.

The inhibition of mitochondrial ETC complexes’ activity by these SCs might be the key player in the neurotoxicity, oxidative stress, and consequent disruption of the mitochondrial bioenergetics balance. Impaired energy metabolism that results in ATP depletion as a consequence of mitochondrial dysfunction has been associated with SC-induced toxicities [36,52,53]. The significantly decreased residual intracellular ATP content relative to the control (Figure 5) in this study supports the fact that butylone, pentylone, and MDPV treatment induced severe cellular energy deficits resulting from mitochondrial impairment in dopaminergic neuronal SH-SY5Y cells. The mechanisms by which these SCs may cause energy impairment remain to be identified. Overall, these data support that a decreased mitochondrial function, specifically the impairment of oxidative phosphorylation through the depletion of energy stores, is critically linked with butylone-, pentylone-, and MDPV-induced neurotoxicity.

Mitochondria also participate in Ca^2+^ buffering, together with the endoplasmic reticulum, in maintaining Ca^2+^ homeostasis. A time- (Figure 6B–D) and dose-dependent (Figure 6E) increase of intracellular Ca^2+^ induced by butylone, pentylone, and MDPV after treatment verified the alteration of intracellular Ca^2+^ regulation. Dysregulated Ca^2+^ release from intracellular reservoirs combined with influx from the extracellular domain could lead to a sustained increase in intracellular Ca^2+^ over time. Meth-mediated neuronal damage involving the disruption of Ca^2+^ homeostasis has been reported [54], but studies on how Ca^2+^ is linked to SC-induced neurotoxicity are scarce. Our work extends the link to SCs disrupting neuronal Ca^2+^ homeostasis that is linked downstream to mitochondrial dysfunction. A further investigation of the exact mechanisms explaining how these SCs contribute to the dysregulation of Ca^2+^ homeostasis, including the identity of protein transporters that are affected, is warranted.

Calcium is involved in many aspects of neuronal physiology, as well as pathophysiology, where Ca^2+^ homeostasis disruption triggered the initiation of the intrinsic apoptotic cell death pathway. The activation of both intrinsic and extrinsic apoptotic pathways has been previously reported for MDPV [36,53], but the death mechanism has remained unknown for both butylone and pentylone. The data obtained in this study indicate that all of the SCs stimulated an apoptotic cell death pathway through the activation of effector caspases 3 and 7. Regardless of the type of activation, the signal sources ultimately activated the common downstream mechanisms, specifically the apoptosis executioner caspase 3 and 7 cascade (Figure 7).

Although the EC_15_ and EC_40_ SC concentrations used in this study are much higher than commonly reported concentrations found in blood (up to 0.09 mM) of post-mortem (PM) cases [19,20,23], they are in good agreement with previously conducted mechanistic in-vitro studies using neuronal cell lines. For example, other researchers have employed ~1.2 and 1.7 mM MDPV in SH-SY5Y cells [36], 1–4 mM 3-FMC in HT22 cells [40], 0.25–20 mM for a series of 13 synthetic cathinones in SH-SY5Y cells [55], and 0.01–2.5 mM MDPV in SH-SY5Y cells [56]. Moreover, PM blood concentrations are usually lower than the peak concentrations expected after the initial drug intake, so are not a useful measure of the maximal drug concentration. Furthermore, it is worth considering that these SCs can accumulate in the intracellular compartment. Hence, it can be assumed that the PM blood levels reported likely underestimate the circulating and intracellular concentrations of SCs. Interestingly, PM findings have shown that the tissue levels of the drug MDMA can be up to 30 times higher in the human brain [57], consistent with the notion that these drugs can traverse the blood–brain barrier (BBB) and accumulate in the brain. Precise prediction of the SC concentration in the brain is unclear and complicated due to the different administration routes and BBB permeabilities of these drugs (which may or may not be compromised in abusers), as well as possible PM SC redistribution [58] and/or metabolism [59]. Moreover, it is important to note that the immortalized dopaminergic neuronal cell line SH-SY5Y can be more resistant to the cytotoxicity of added drugs. One possibility of the more resistant differentiated SH-SY5Y cellular phenotype may be linked to the up-regulated expression of Nrf2 proteins that provide an enhanced capacity to buffer the oxidative insults [60]. Hence, the pathophysiological outcomes from this study are tempered by the drug dose required to elicit responses in this cell culture model. While it is unclear precisely how the actual dose of SCs used in this study is pharmacologically or even clinically relevant, or indeed whether the dose range employed in this work can be translated to the human usage of recreational SCs, particularly at the time of administration, it remains important to understand the type of damage elicited in different cell types that may be vulnerable to these drugs. This, in turn, may provide some understanding on the potential for similar doses in humans (if achievable) to cause neuronal cell damage via mechanisms that involve mitochondrial dysfunction leading to a decreased cell viability. For the above reasons, the neurotoxicity findings observed in this in-vitro system should be repeated in cultures of primary neuronal cell cultures and in-vivo models to ascertain the sensitivity to a lower SC dose as a further model of neuronal toxicity for these drugs.

The chronic use of psychostimulants has been associated with cognitive dysfunction and various neuropsychological consequences, such as impaired impulse control, working memory, decision making, attention, and motor coordination [32,61,62]. In addition, meth use has been linked to an increased risk of developing neurodegeneration diseases, such as Parkinson’s disease (PD) [63]. Repeated long-term acute exposure of meth has been associated with the neurodegeneration of dopaminergic neurons, reflecting the degeneration pattern observed in PD patients [64]. These epidemiological findings suggest that only a very small proportion of meth users are subsequently diagnosed with PD and the usage of dopamine substitution therapy among meth users is still in a premature investigational stage [65]. Given the chemical structure similarities of the SCs to meth, it is reasonable to postulate that long-term high-dosage exposure of these SCs might also result in Parkinson-like development. Reports have shown that SC users have also been associated with homelessness [66] or lower socioeconomic areas [67], where a greater risk of poor health with a poor antioxidant system further enhanced their susceptibility to SC toxicity. Behavioral factors such as physical exercise [68] and healthy dietary lifestyle measures via an increased anti-oxidative capacity have been shown to be a promising approach in the management of psychostimulant addiction [69]. There is currently no remedy for SC intoxication, except for supportive care [70]; however, the oxidative stress mechanistic pathway suggests the possibility of efficacious antioxidant pharmacotherapies for attenuating SC-induced ROS production [71]. Considering that mitochondrial dysfunction plays a critical part in SC-induced neurotoxicity, the enhancement of mitochondrial function [72] and mitochondrial transplantation [73] have received attention as strategies that might ameliorate the associated mitochondrial neurodegeneration. Moreover, the development of therapeutic strategies that stabilize neuronal Ca^2+^ homeostasis by targeting the calcium channels may also be capable of preventing neurotoxicity induced by these SCs [74].

## 4. Materials and Methods

### 4.1. Chemical Synthesis

Hydrochloride salts of butylone, pentylone, and MDPV were synthesized in-house with a purity greater than 98%. The synthesis of salts involved a multiple-step reaction sequence: Grignard reaction of piperonal, oxidation, α-bromination, and nucleophilic substitution with methylamine (for butylone and pentylone HCl) and pyrrolidine (for MDPV HCl). The final product salt was obtained following extraction and HCl work-up. The salts were fully characterized by proton (^1^H-NMR) and carbon (^13^C-NMR) nuclear magnetic resonance spectroscopy, gas chromatography-mass spectrometry (GC-MS), Fourier-transform infrared (FTIR) spectroscopy, and ultraviolet (UV) spectroscopy. Butylone HCl: ^1^H-NMR (500 MHz, D_2_O) δ, ppm: 7.705–7.686 (dd, ^3^*J*_HH_ = 8.25 Hz, ^4^*J*_HH_ = 1.25 Hz, 1H), 7.481–7.478 (d, ^4^*J*_HH_ = 1.5 Hz, 1H), 7.045–7.028 (d, ^3^*J*_HH_ = 8.5 Hz, 1H), 6.134–6.130 (d, ^2^*J*_HH_ = 2.0 Hz, 2H), 5.025–5.003 (t, ^3^*J*_HH_ = 5.5 Hz, 1H), 2.741 (s, 3H), 2.131–2.040 (m, 2H), 0.889–0.859 (t, ^3^*J*_HH_ = 7.5 Hz, 3H); ^13^C–NMR (125 MHz, CD_3_OD) δ, ppm: 194.114, 153.006, 148.401, 128.603, 125.986, 108.719, 107.987, 102.687, 62.641, 31.494, 23.187, 8.385; EI (*m/z*, %): 221(M^+^,0), 219(2), 149(11), 121(7), 72(100), 70(30); FTIR (ATR) ν_max_, cm^−1^: 2909 (C-H stretch), 2689 and 2475 (asym. and sym. NH_2_^+^ stretches), 1674 (C=O stretch), 1602 and 1452 (C=C stretch), 1252 (C-C=O stretch), 1035 (C-O stretch), 880 and 802 (1,2,4-trisubstituted benzene); UV (H_2_O) λ_max_, nm: 236, 282, 321. Pentylone HCl: ^1^H-NMR (500 MHz, D_2_O) δ, ppm: 7.696–7.679 (d, ^3^*J*_HH_ = 7.5 Hz, 1H), 7.467 (s, 1H), 7.040–7.023 (d, ^3^*J*_HH_ = 8.5 Hz, 1H), 6.129 (s, 2H), 5.024–5.002 (t, ^3^*J*_HH_ = 5.5 Hz, 1H), 2.734 (s, 3H), 2.031–1.916 (m, 2H), 1.365–1.218 (m, 2H), 0.874–0.845 (t, ^3^*J*_HH_ = 7.25 Hz,3H); ^13^C-NMR (125 MHz, CD_3_OD) δ, ppm: 194.334, 153.024, 148.416, 128.728, 126.008, 108.726, 107.979, 102.706, 61.799, 32.142, 31.566, 17.295, 13.855; EI (*m/z*, %): 235(M^+^,0), 192(24), 164(8), 149(100), 121(20), 91(4), 65(9); FTIR (ATR) ν_max_, cm^−1^: 2963 (C-H stretch), 2744 and 2481 (asym. and sym. NH_2_^+^ stretches), 1675 (C=O stretch), 1603 and 1453 (C=C stretch), 1256 (C-C=O stretch), 1033 (C-O stretch), 863 and 806 (1,2,4-trisubstituted benzene); UV (H_2_O) λ_max_, nm: 236, 282, 321. MDPV HCl: ^1^H-NMR (500 MHz, D_2_O) δ, ppm: 7.725–7.709 (d, ^3^*J*_HH_= 8.0 Hz, 1H), 7.496 (s, 1H), 7.061–7.044 (d, ^3^*J*_HH_ = 8.5 Hz, 1H), 6.148–6.142 (d, ^4^*J*_HH_ = 3.0 Hz, 2H), 5.122 (br. s, 1H), 3.455–3.284 (m, 4H), 2.128–1.999 (m, 6H), 1.248–1.147 (m, 2H), 0.850–0.821 (t, ^3^*J*_HH_ = 7.25 Hz, 3H); ^13^C-NMR (125 MHz, CD_3_OD) δ, ppm: 194.334, 153.024, 148.416, 128.728, 126.008, 108.726, 107.979, 102.706, 61.799, 32.142, 31.566, 24.389, 17.295, 13.855; EI (*m/z*, %): 275(M^+^,0), 149(5), 126(100), 96(4), 55(5); FTIR (ATR) ν_max_, cm^−1^: 2969 (C-H stretch), 2610 (NH^+^ stretch), 1684 (C=O stretch), 1609 and 1435 (C=C stretch), 1253 (C-C=O stretch), 1033 (C-O), 867 and 807 (1,2,4-trisubstituted benzene); UV (H_2_O) λ_max_, nm: 237, 285, 324.

### 4.2. Cellular Studies

The Countess^TM^ cell counting chamber slide and TB Stain 0.4% *w*/*v* were purchased from Invitrogen (Eugene, OR, USA). The LDH cytotoxicity assay kit was purchased from Cayman Chemical (Ann Arbor, MI, USA). The DCFDA/H2DCFDA (2′,7′-dichlorofluorescin diacetate)—Cellular reactive oxygen species (ROS) detection assay kit and Cal-520 AM were purchased from Abcam (Cambridge, UK). Seahorse Extracellular Flux (XF) 96 cell culture microplates, the XF 96 assay kit, the Cell Mito Stress Test Kit, XF calibrant, XF Base Medium, and the XF96 Analyzer were obtained from Seahorse Bioscience (North Billerica, MA, USA). The ATPlite luminescence assay system was purchased from Perkin Elmer (Waltham, MA, USA). The Caspase-Glo^®^ 3/7 assay was purchased from Promega (Madison, WI, USA). T75 tissue culture flasks, and 96-well and 6-well cell culture plates were purchased from Corning (Corning, NY, USA). The phosphate buffered saline (PBS) tablet, copper (II) sulfate solution (CuSO_4_, 4% *w*/*v*), bicinchoninic acid (BCA), phorbolester 12-*O*-tetradecanoylphorbol-13-acetate (TPA), retinoic acid (RA), and all cell culture materials were purchased from Sigma-Aldrich (St. Louis, MO, USA). Milli-Q water (18.2 MΩ cm^−1^) was obtained from Sartorius (Göttingen, Germany). The Countess™ II automated cell counter was purchased from Thermo Fisher Scientific (Waltham, MA, USA). The Axio Vert.A1 inverted microscope was purchased from Zeiss (Jena, Germany). Centrifuge 5415 R and 5810 R were purchased from Eppendorf (Hamburg, Germany). The Orbit P4 digital microtube and microplate orbital shaker was obtained from Labnet (Edison, NJ, USA). The IncuCyte^®^ system was purchased from Essen BioScience (Ann Arbor, MI, USA). The Infinite^®^ M200 Pro plate reader was purchased from Tecan (Männedorf, Switzerland).

### 4.3. Cell Culture

The human neuroblastoma SH-SY5Y cell line (ATCC, Manassas, VA, USA) was grown in a T75 tissue culture flask containing Dulbecco’s Modified Eagle’s Medium (DMEM)/F-12 Ham mixtures supplemented with 10% heat-inactivated fetal bovine serum (HI-FBS), l-glutamine (2 mM), a mixture of penicillin/streptomycin (100 U/mL/100 μg/mL), and non-essential amino acid solution (NEAA, 1×). Cells were cultured at 37 °C in a humidified atmosphere incubator containing 95% air and 5% CO_2_, until 75–80% confluence. Media was changed every 2 to 3 days. Where required, cells were harvested by subculture with media containing trypsin/EDTA (0.12% trypsin/ 0.02% EDTA *w*/*v*). The cells were passaged over 8 passages (Passage 6–13) and counted under TB staining using an automated cell counter. Cells were seeded in full media (10% HI-FBS) onto multi-well plates overnight at the required density. Cells were differentiated to a dopaminergic neuronal phenotype by a previously reported method, with slight modification [75], using 10 µM RA media (2.5% HI-FBS) for 3 days, followed by a mixture of 10 µM RA and 81 nM TPA (2.5% HI-FBS) for another 3 days. General cell morphology was monitored at regular intervals using phase contrast inverted microscopy.

### 4.4. Cytotoxicity Assays

Cells were sub-cultured and seeded at a density of 25,000 cells/cm^2^. Cells were then treated with butylone, pentylone, and MDPV individually for 24 h at 37 °C. This exposure time was identical to those used for other in-vitro studies involving similar SC doses [36,40,56,76,77]. Dose-response curves for these SCs (1 to 10 mM) were determined using TB and LDH assays. The effective concentrations of EC_15_, EC_40_, and EC_50_, that is, the concentration that induces 15%, 40%, and 50% cell death, respectively, for each drug were determined from the dose-response curves. The calculated mean EC_15_ and EC_40_ of butylone, pentylone, and MDPV (refer to tabulated data in Figure 1) were then used for subsequent experiments.

### 4.5. TB Assay

Upon treatment, drug media containing non-adherent cells was transferred from the 6-well plate into a 1.5 mL tube and centrifuged (200 relative centrifugal force, r.c.f; 6 min). Adherent cells were harvested as described above and resuspended into the pellet cells obtained from the media fraction. The total number of cells were then counted using an automated cell counter. Data were obtained from three independent experiments. Background subtraction (untreated control) was performed for all wells. Finally, the % cytotoxicity was calculated as the percentage of the ratio of non-viable cells to the total number of cells.

### 4.6. LDH Assay

Upon treatment, a 96-well plate was centrifuged (400 r.c.f; 5 min). Next, 100 µL of the isolated supernatant was transferred to another 96-well plate and each well was treated with 100 µL of reaction mixture containing nicotinamide adenine dinucleotide (NAD^+^), lactic acid, iodonitrotetrazolium salt (INT), and reconstituted diaphorase, prepared according to the manufacturer’s instruction. Treated samples were incubated at 37 °C with shaking on an orbital shaker. After 30 min, absorbance was measured at 490 nm with a Tecan plate reader. Data were obtained from four independent experiments with background subtraction (media only) prior to determination of the % cytotoxicity, as follows (Equation (1) below):

% Cytotoxicity of sample = (A_sample_ − A_assay buffer_)/(A_Triton X−100_ − A_assay buffer_) × 100. (1)

### 4.7. Measurement of Intracellular ROS

Levels of intracellular ROS were determined using a commercial kit, as per the manufacturer’s instruction. Briefly, cells were seeded at a density of 50,000 cells/cm^2^ onto a 96-well black, clear-bottom plate. Cells were rinsed with 1× buffer (provided in the kit) and incubated with 100 µL DCFDA (25 µM) at 37 °C for 45 min in the dark. Cells were rinsed with 1× buffer before being treated with each drug individually. Fluorescence was measured at Ex/Em: 485/535 nm with a Tecan plate reader at different time points (2, 4, 6, and 24 h). Background subtraction (media only) was performed for all wells. Data were obtained from four independent experiments and the data was expressed as a % fold-change relative to the control.

### 4.8. Measurement of Mitochondrial Respiration

Cells were seeded at a density of 20,000 cells/80 µL well onto XF 96-well cell culture microplates. Upon the individual treatment of each drug, media was replaced with XF base media with added glucose (10 mM), sodium pyruvate (1 mM), and L-glutamine (2 mM), and kept in a non-CO_2_ incubator at 37 °C for 1 h. Analysis of OCR was performed in a Seahorse XF96 analyzer, according to the manufacturer’s instructions. Mitochondrial respiration kinetics using the Seahorse analyzer was employed to provide valuable information regarding the extent of mitochondrial dysfunction in response to butylone, pentylone, and MDPV. The measurement of OCR using the Seahorse analyzer has been proven to be the most widely used technique in determining mitochondrial bioenergetics dysfunction in cells [78]. OCR was measured in a basal condition, and following the injection of each compound: ATP synthase inhibitor oligomycin (1 µM), the mitochondrial uncoupler FCCP (0.5 µM), and a mixture of complex I + II inhibitors rotenone with antimycin A (1 µM + 1 µM). The mixture was incubated for 3 min, followed by a 3 min lag-time for each cycle. Three measurements were taken for each condition. Data were obtained from at least four independent experiments and normalized to % confluence using the IncuCyte^®^ system and standard software.

### 4.9. Measurement of Intracellular Adenosine Triphosphate (ATP)

Levels of intracellular ATP were determined using a commercial kit, as per the manufacturer’s instructions. Briefly, cells were seeded at a density of 25,000 cells/cm^2^ onto a 96-well plate. Following individual drug treatment, media was replaced with a mixture of 100 µL of PBS (0.01 M, pH 7.5) and 50 µL of mammalian cell lysis solution (provided in the kit). The mixture was mixed in an orbital shaker (700 revolutions per min (r.p.m.); 5 min). In the dark, 50 µL of substrate, luciferase-lucerin (provided in the kit), was added and shaken for another 5 min at 700 r.p.m. The plate was left in the dark (10 min; 22 °C) and luminescence was measured with a Tecan plate reader. Intracellular ATP levels were determined with a calibration curve that was obtained in parallel on the same plate using a 10 mM stock solution of reconstituted lyophilized ATP standard prepared in Milli-Q water. Data were obtained from three independent experiments and normalized to the total protein using the BCA assay method. Briefly, protein levels in the cell lysates were measured with 190 µL of CuSO_4_/BCA solution (0.02% *v*/*v*) added to each well containing 10 µL of BSA standards or treated samples (5 µL of Milli-Q water was added to 5 µL of sample). The mixture was incubated (37 °C; 25 min) and absorbance was measured at 562 nm with a Tecan plate reader. Values were standardized against a BSA protein standard curve generated on the same plate.

### 4.10. Real-Time Imaging and Measurement of Intracellular Ca^2+^

Cells were seeded at a density of 25,000 cells/cm^2^ onto a 96-well black, clear-bottom plate. Cells were incubated with 100 µL of Cal-520 AM (5 µM) at 37 °C for 1 h and then allowed to equilibrate to 22 °C for a further 30 min before treatment of the cells with individual drugs. Fluorescence was measured at Ex/Em: 490/525 nm with a Tecan plate reader. Background subtraction (media only) was performed for all wells. Data were obtained from four independent experiments and the data was expressed as a % fold-change relative to the control. Phase contrast and fluorescence images of cellular Ca^2+^ were also acquired using a real-time imaging IncuCyte^®^ system at different time points (8, 12, and 22 h). Cal-520 AM was excited with a 440–490 nm filter and emitted fluorescence was collected with a 504–544 nm filter (green channel). Images were captured using a 10x objective.

### 4.11. Measurement of Caspase 3 and 7 Activity

Cells were seeded at a density of 25,000 cells/cm^2^ onto 96-well white, clear-bottom plates. Upon the individual treatment of each drug, media was replaced with 100 µL of 2.5% HI-FBS media. The plate was allowed to equilibrate at 22 °C and 100 µL of Caspase-Glo^®^ 3/7 reagent was then added and the plate was incubated in the dark. The mixture was mixed in an orbital shaker (500 r.p.m., 30 s) and further incubated at 22 °C for 1.5 h. Luminescence was measured with a Tecan plate reader. Background subtraction (media only) was performed for all wells. Data were obtained from four independent experiments and normalized to % confluence using the IncuCyte^®^ system employing standard software.

### 4.12. Data Analysis

Data was expressed as the mean ± standard deviation from at least three independent experiments. For comparisons, data were analysed using one-way analysis of variance (ANOVA) followed by Tukey’s test using Graph Pad Prism 7. Statistical significance was set at *p* < 0.05. EC_50_ values were obtained from the dose-response curve. EC_15_ and EC_40_ values were calculated using Graph Pad QuickCalcs. The XF mito stress test report generator automatically calculated the XF cell mito stress test parameters from wave data that were exported to Excel.

## 5. Conclusions

The present study sheds light on the cellular and molecular mechanisms underlying the neurotoxicity potency of butylone, pentylone, and MDPV. The apoptotic cell death pathway implicated the orchestration of mitochondrial-mediated toxicity mechanisms via oxidative stress, a compromised bioenergetics balance, and changes in Ca^2+^ homeostasis. Understanding the mechanisms underlying these neurotoxic actions and related clinical manifestation is imperative in the management and treatment of acute-neurological complications arising from SC addiction. The potential clinical relevance of this study includes the demonstration that butylone, pentylone, and MDPV (i) trigger neuronal apoptosis and stimulate (ii) increased intracellular Ca^2+^ and (iii) mitochondrial dysfunction, leading to a loss in ATP, which is information that may be useful in understanding the detrimental impact of these drugs. The findings in this study should be viewed as a model for understanding the neurotoxic mechanisms that may play a role in the response of the human central nervous system to SC exposure. Substantial work remains before safe and novel therapeutic agents may be developed and translated for human use in the prevention of the devastating neurotoxic effects associated with the abuse of these drugs.
